# Ethanol (E) Impairs Fetal Brain GSH Homeostasis by Inhibiting Excitatory Amino-Acid Carrier 1 (EAAC1)-Mediated Cysteine Transport

**DOI:** 10.3390/ijms18122596

**Published:** 2017-12-05

**Authors:** Dhyanesh Patel, Lenin Mahimainathan, Madhusudhanan Narasimhan, Marylatha Rathinam, George Henderson

**Affiliations:** Department of Pharmacology and Neuroscience, Texas Tech University Health Sciences Center, 3601 4th Street, Lubbock, TX 79430, USA; dhyanesh.patel@ttuhsc.edu (D.P.); madhu.narasimhan@ttuhsc.edu (M.N.); lathalenin@yahoo.com (M.R.); george.henderson@ttuhsc.edu (G.H.)

**Keywords:** ethanol, fetal, brain, neuron, glutathione, cysteine, EAAT3/EAAC1/*Slc1a1*

## Abstract

Central among the fetotoxic responses to in utero ethanol (E) exposure is redox-shift related glutathione (GSH) loss and apoptosis. Previously, we reported that despite an E-generated Nrf2 upregulation, fetal neurons still succumb. In this study, we investigate if the compromised GSH results from an impaired inward transport of cysteine (Cys), a precursor of GSH in association with dysregulated excitatory amino acid carrier1 (EAAC1), a cysteine transporter. In utero binge model involves administration of isocaloric dextrose or 20% E (3.5 g/kg)/ by gavage at 12 h intervals to pregnant Sprague Dawley (SD) rats, starting gestation day (gd) 17 with a final dose on gd19, 2 h prior to sacrifice. Primary cerebral cortical neurons (PCNs) from embryonic day 16–17 fetal SD rats were the in vitro model. E reduced both PCN and cerebral cortical GSH and Cys up to 50% and the abridged GSH could be blocked by administration of *N*-acetylcysteine. E reduced EAAC1 protein expression in utero and in PCNs (*p* < 0.05). This was accompanied by a 60–70% decrease in neuron surface expression of EAAC1 along with significant reductions of *EAAC1/Slc1a1* mRNA (*p* < 0.05). In PCNs, EAAC1 knockdown significantly decreased GSH but not oxidized glutathione (GSSG) illustrating that while not the sole provider of Cys, EAAC1 plays an important role in neuron GSH homeostasis. These studies strongly support the concept that in both E exposed intact fetal brain and cultured PCNs a mechanism underlying E impairment of GSH homeostasis is reduction of import of external Cys which is mediated by perturbations of EAAC1 expression/function.

## 1. Introduction

**Figure 1:**
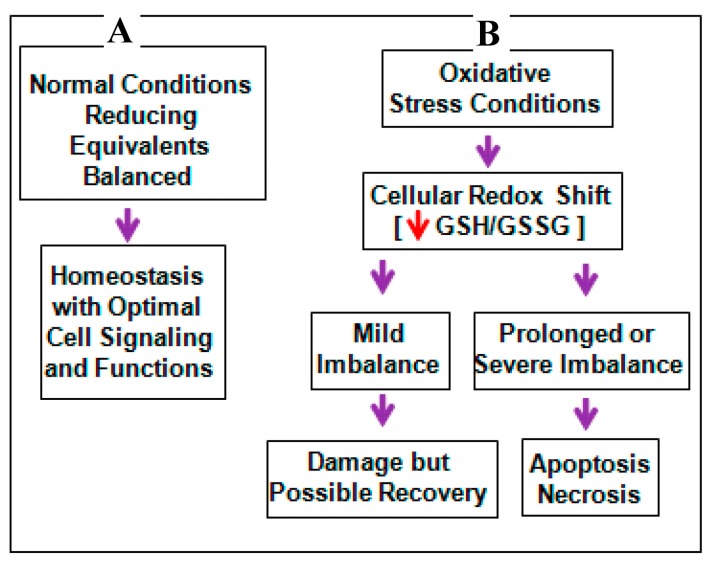
Under normal physiological conditions, redox homeostasis is maintained by a balance between the reactive oxygen species (ROS) and antioxidant components, mainly GSH/GSSG couple that acts as reducing equivalents leading to normal cellular signaling (**A**); when cells encounter mild stress, an imbalance in the redox shift marked by diminished GSH/GSSG ensues. Whilst, an appropriate management and/or interventions can normalize this shift and can rescue the cellular system against potential toxicity (**B**). However, during intense stress challenge, a toxic level of ROS can accumulate shifting the redox balance that cannot be reversed, leading to macromolecule damage (**B**). Red arrow indicates decrease in the levels and purple arrow indicates the flow of events.

Fetotoxic effects of maternal ethanol (E) intake are well documented in animals and humans. Central among the responses to E exposure are developmental deficits affecting multiple brain regions, including the cerebellum, hippocampus, olfactory bulbs, and cerebral cortex [1,2,3,4]. In vivo, moderate, clinically relevant E can generate an enhanced apoptotic loss of neurons in the developing brain [4,5,6,7,8,9,10,11]. E is prooxidant and fetal cells are highly sensitive to E/oxidative stress (OS) [5,12,13,14]. Thus, redox imbalance is a mechanism underlying E-induced neuron apoptosis in developing brain [5,12,13,14]. E produces an enhanced apoptotic death in a subpopulation of cells with the lowest glutathione (GSH) content and normalizing this redox imbalance by stabilizing neuron GSH content prevents the neuron death [5]. Thus, a mechanism underlying E-induced fetal neuron apoptotic death is OS which has propelled research into what is now called “redox control of teratogenesis” [15,16] or the “redox hypothesis” (Figure 1). This “redox hypothesis” is a disruption of cellular redox circuits which are maintained by thiol disulfide “couples” including reduced/oxidized glutathione (GSH/GSSG) and cysteine/cystine (Cys/CySS) [17,18]. It occurs in the E-exposed developing brain [5,12,19,20] with redox shifts that are subject to complex interactive neuroprotective responses impacted by E. GSH and thioredoxin (TRX) both regulate the thiolylation and dithiol/disulfide redox switches thereby controlling transition into apoptosis. In this regard, OS is a critical driver of teratogenesis and teratogenesis elicited by E occurs by induction of oxidative damage that disrupts stress pathways [21,22,23,24]. 

A/the central regulator of GSH homeostasis, hence redox balance mitigation of neuron apoptotic death [5], is the master transcription factor, nuclear factor E2-related factor 2 (Nrf2). Nrf2 regulated genes include glutamate-cysteine ligase (GCL) (rate limiting enzyme in GSH synthesis), glutathione reductase (generation of reduced/active GSH), and multiple components of the γ glutamyl cycle by which the GSH synthesis substrate, Cys is supplied to neurons in addition to dietary source(s) [20,25]. Previous study in our laboratory addressed Nrf2/ARE (antioxidant response element)-mediated neuroprotection which should prevent neuron death by maintaining GSH homeostasis [7]. E does up-regulate Nrf2 and the knockout of Nrf2 illustrated that this increase in Nrf2 confers a certain degree of neuroprotection [7]. However, somehow E prevents Nrf2 from providing complete protection and a population of fetal neurons still succumbs unless we artificially increase Nrf2 beyond its innate capacity [7].

**Figure 2:**
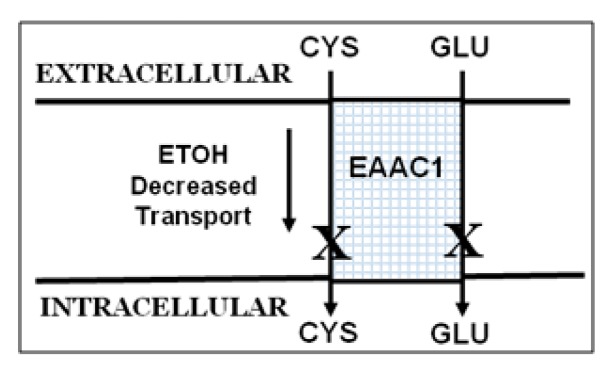
EAAC1—a neuronal transporter for cysteine (Cys) and glutamate (Glu).

A potential explanation for this incomplete protection resides in the kinetics of the GSH synthesis path. GSH, a tripeptide is synthesized in a two-step enzymatic reaction [26,27] with GCL ultimately regulated by the availability of Cys [28,29]. Cys availability is a critical control point for GSH synthesis [30]. Current thinking is that about 90% of neuron Cys uptake from extracellular supplies is by sodium-dependent Glu transporters, mainly by the excitatory amino acid transporter, EAAT3/EAAC1 [26,31,32,33]. Inward transport of Cys and Glu is a 1:1 ratio (Figure 2). EAAC1 affinity for Cys is about equal to that for Glu and EAAC1 KO mice show a 40% decrease in GSH [34,35], along with sensitivity to brain damage not connected to Glu neurotoxicity that can be blocked by normalizing brain GSH [34,36,37,38,39].

Since, during the early-E sensitive stages of brain development, neurons maintain GSH in the absence of mature astrocytes (AST) or oligodendroglia and given the causal association of OS with GSH loss in alcohol-induced neurodevelopmental toxicity, it is critical to determine the origin(s) of E-related GSH loss. Thus, in the current study, we tested if (a) E-induced GSH loss occurs due to less availability of its obligate precursor, cysteine; and (b) if so, does the insufficient cellular supply of cysteine result from an aberrant EAAC1-dependent cysteine uptake into the fetal neurons?

## 2. Results

### 2.1. Ethanol Decreases Cysteine (Cys) and Glutathione (GSH) in Primary Cerebral Cortical Neurons (PCNs) and Fetal Brains

**Figure 3:**
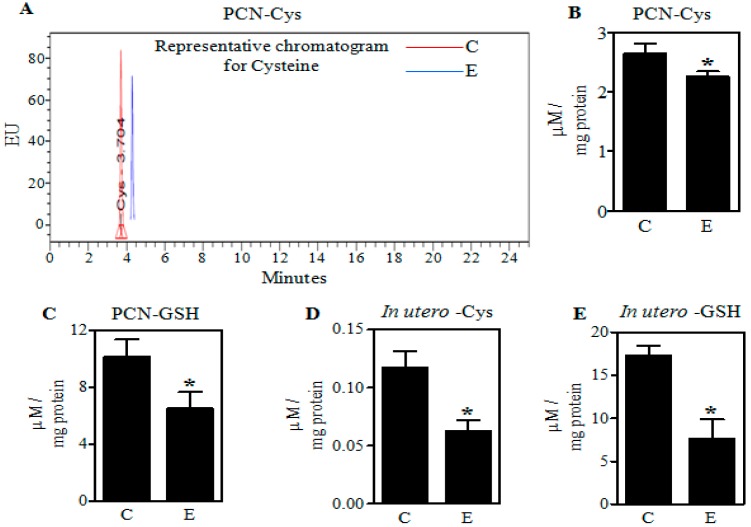
Effect of ethanol on Cys and GSH levels in PCNs and fetal brain cortices. A representative high pressure liquid chromatography (HPLC) profile of Cys in Control (C) and ethanol (E)-treated PCNs (**A**); The concentration of Cys quantified using standards in PCNs (*n* = 4) (**B**); Quantification of GSH concentration in control and E-treated primary cerebral cortical neurons (PCNs) using standards as measured by HPLC (*n* = 4) (**C**); HPLC-based determination of Cys concentration in fetal brain cortices of binge alcohol-exposed pregnant rats (*n* = 7) (**D**); Fetal brain cortex GSH content following binge alcohol gestational exposure using HPLC (*n* = 4) (**E**). Values represent the mean ± SEM. * *p* < 0.05 was considered significant for ethanol alone.

Fetal rat cerebral cortical neurons were treated with E (4 mg/mL) for 24 h and pregnant dams were treated with the “Binge” model as detailed above. Both of these regimens elevate Nrf2 expression as well as induce enhanced apoptotic death of neurons [5,7]. To gain a better understanding of the E-induced GSH loss, we first assessed the levels of cysteine, which is one of the key substrates involved in the de novo synthesis of GSH. Illustrated in Figure 3, both in vivo maternal exposures and the in vitro PCNs treatment with E reduced Cys with a concomitant decrease in the GSH content. Cys was decreased in PCNs and fetal cerebral cortices by 16% and 48% (*p* < 0.05), respectively (Figure 3A–C). These responses correlated with 36% and 55% (*p* < 0.05) reductions in GSH in PCN and cerebral cortices (Figure 3D,E).

### 2.2. Ethanol Decreases Excitatory Amino Acid Carrier1 (EAAC1) Protein and Its Membrane Expression

**Figure 4:**
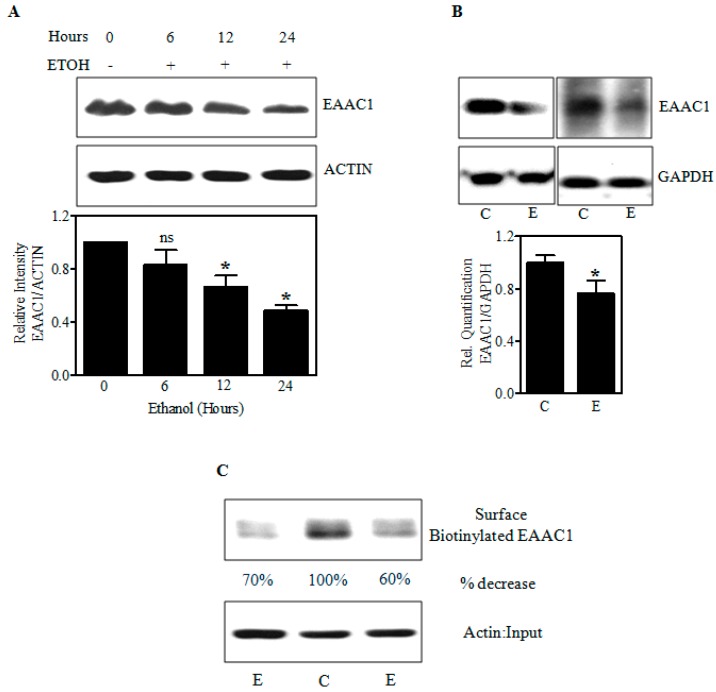
Effect of ethanol on the cellular and surface expression of the EAAC1 protein. Immunoblot analysis for EAAC1 and ACTIN or glyceraldehydes-3-phosphate dehydrogenase (GAPDH) in Control and E-treated PCNs (*n* = 4) (**A**); in fetal brain cortices obtained from in utero iso-caloric dextrose or alcohol-exposed pregnant dams (*n* = 6) (**B**); Biotinylation of cell surface proteins from control and E-exposed PCNs was processed for biotinylation assay as in Materials and Methods. Biotinylated cell surface EAAC1 and the corresponding unbiotinylated intracellular Actin were analyzed by Western blot with indicated antibodies (**C**); For C, the percentage decrease is obtained from control cells with *n* = 2 pooled from 3 individual samples. * *p* < 0.05 was considered significant for ethanol alone.

As in vitro and in utero E decreased the cysteine levels, we next determined whether this was associated with altered expression of EAAC1, a chief player in cysteine transport and uptake into the cells [26,32,33]. The immunoblot in Figure 4A shows that E progressively decreased EAAC1 protein in fetal PCNs by 36% and 50% (*p* < 0.05) within 12 and 24 h, respectively. Also, the 2 days in utero E binge decreased the protein expression of EAAC1 in fetal brain cerebral cortices by 25% (*p* < 0.05) (Figure 4B). Since E decreased EAAC1 protein levels, we investigated whether this is reflected in the expression of EAAC1 transporter at the neuronal cell surface by an in vitro biotinylation assay. Determinations of E effects on EAAC1 in the plasma membrane are key studies as their external expression is directly linked to its transporter function [40,41,42]. Biotinylated EAAC1 detected by immunoblot represents the plasma membrane steady-state levels excluding endocytosed EAAC1 [43] and the EAAC1 immunoreactivity was strikingly decreased (60% to 70%) in the biotinylated fraction of E-treated neurons (Figure 4C). Equal levels of actin signal in the input denoted that similar numbers of C and E neurons were used in the assay. These data suggest that E can impair EAAC1 protein expression and its surface presentation likely reflecting reduced Cys transport by this system.

### 2.3. Ethanol-Induced Reduction of EAAC1 Protein Is Associated with a Decrease in Its Transcript Levels

**Figure 5:**
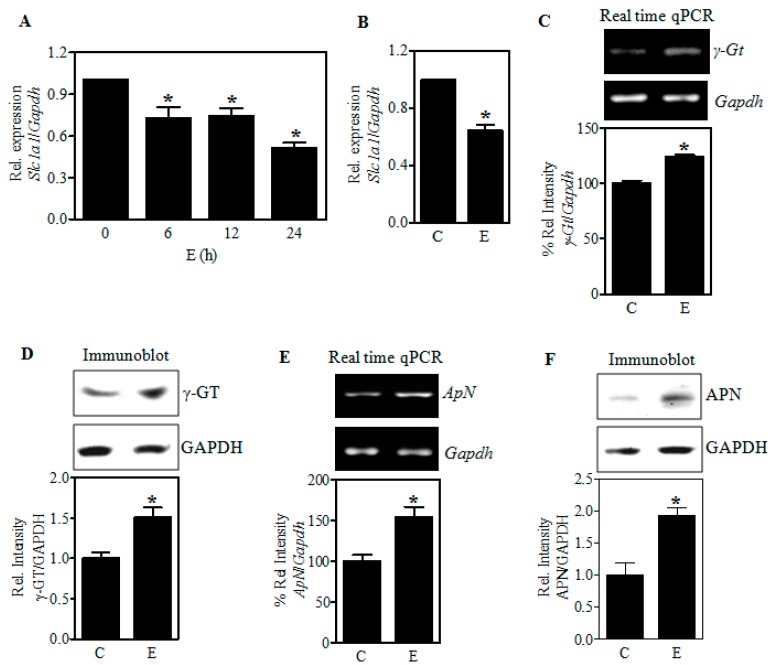
Effect of ethanol on the expression of EAAC1, γ-glutamyl transpeptidase (γ-GT), and Aminopeptidase N (APN). PCNs were treated with E (4 mg/mL) for the periods indicated and real-time qRT-PCR analysis for *Slc1a1/EAAC1* transcript expression was performed. The fold change expression of *Slc1a1* was determined by normalizing with the expression of a housekeeping gene, *Gapdh* (*n* = 3) (**A**); Pregnant rats (Sprague-Dawley) at embryonic day 17 were administered 5 doses of E (3.5 g/kg b.wt.) or isocaloric dextrose by gastric intubation at 12-h intervals. At embryonic day 19 brain cortex from embryos were dissected and processed for *Slc1a1* and *Gapdh* gene expression by qRT-PCR as in panel A, (*n* = 5) (**B**); Fetal brain cortices obtained as shown in Panel B was subjected to one step RT-PCR for *γ-Gt* (*n* = 6) (**C**); and *ApN* mRNA (*n* = 6) (**E**) expression. The top panels in C and E indicate a representative ethidium bromide staining image for *γ-Gt* and *ApN* mRNA expression respectively with *Gapdh* mRNA expression serving as the loading control. The bottom panels represent the percentage relative change of the respective targets normalized to *Gapdh* mRNA levels from control and E-treated fetal brain cortices. Protein extracts from fetal brain cortices were analyzed by Western blotting with antibodies for γ-GT (*n* = 3) (**D**); and APN (*n* = 3) (**F**). Top panels show a representative blot and the bottom panel shows the γ-GT and APN normalized to GAPDH protein intensity. Values represent the mean ± SEM. * *p* < 0.05 was considered significant for ethanol alone.

To assess whether the E-induced *Slc1a1* dysregulation occurs at the transcriptional level, we performed real-time qPCR analysis for *Slc1a1* mRNA expression in E treated PCN and in fetal cerebral cortices exposed to E in utero. Figure 5A illustrates that E exposure (4 mg/mL) reduced the *Slc1a1* transcript expression levels by 27% (*p* < 0.05) as early as 6 h in PCNs. This was further reduced to 49% (*p* < 0.05) at the end of a 24 h exposure (Figure 5A). Similarly, a significant decrease by about 36% (*p* < 0.05) in the *Slc1a1* mRNA expression was observed in the fetal brain neocortices obtained from in utero binge exposed pregnant rats (Figure 5B). These results support the concept that ethanol-induced downregulation of EAAC1 could reflect impairment at either the transcriptional or post-transcriptional level.

### 2.4. Ethanol Does Not Restrict the Expression of γ-Glutamyl Transpeptidase (γGT) and Aminopeptidase N (APN), the Extracellular GSH-Cleaving Cysteine Homeostasis Factors

Next, we determined if the Cys unavailability is due to loss and/or dysregulation of GSH-cleaving enzymes, *γGt*, and *ApN* that supply Cys to be transported into the cells. One-step RT-PCR analysis for the expression of *γGt* and *ApN* mRNA expression normalized to *Gapdh* showed that in utero E did not decrease, but rather increased the expression of these genes significantly (*p* < 0.05) in the fetal cerebral cortices (Figure 5C,E). In consistent with the mRNA changes, E significantly increased the protein expression of γGT and APN in fetal brain cortices (Figure 5D,F). This suggests that the important regulatory enzymes involved in GSH breakdown to cysteine have been enhanced as would be expected to provide adequate extracellular Cys supply under stress.

### 2.5. Inhibition of EAAC1 Expression (as by E) Reduces Neuron GSH Synthesis

**Figure 6:**
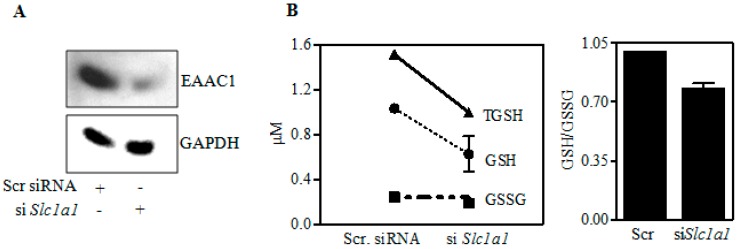
Effect of siRNA-mediated *Slc1a1* knockdown on GSH and GSSG levels. PCNs were transfected with either nontargeting scramble siRNA or a SMARTpool mix of four si*Slc1a1* using siPORT amine. 48 h post-transfection, protein extracts were immunoblot-analyzed for EAAC1 (**A**) (*n* = 3); and fluorimetry-based determination of GSH and GSSG content (**B**). The bar graph represents the GSH/GSSG ratio with *n* = 2 pooled from 3 individual samples (**B**).

Since E-reduced the expression of EAAC1 and associated GSH, we probed the role of EAAC1 in regulation of GSH. To this end, we silenced EAAC1 level in PCNs. Figure 6A illustrates a 70% knockdown of EAAC1 in PCNs. This knockdown reduced GSH by 22% (Figure 6B) showing that EAAC1 transporter plays an important role in neuron GSH homeostasis. In addition, the decline in GSH with no effect on GSSG illustrates that the effect was on GSH synthesis. These experiments further support a role for an E-related inhibition of EAAC1 in the observed reduction of neuronal Cys and GSH.

### 2.6. Increasing Fetal Brain Cys Prevents the Ethanol-Mediated GSH Reduction

**Figure 7:**
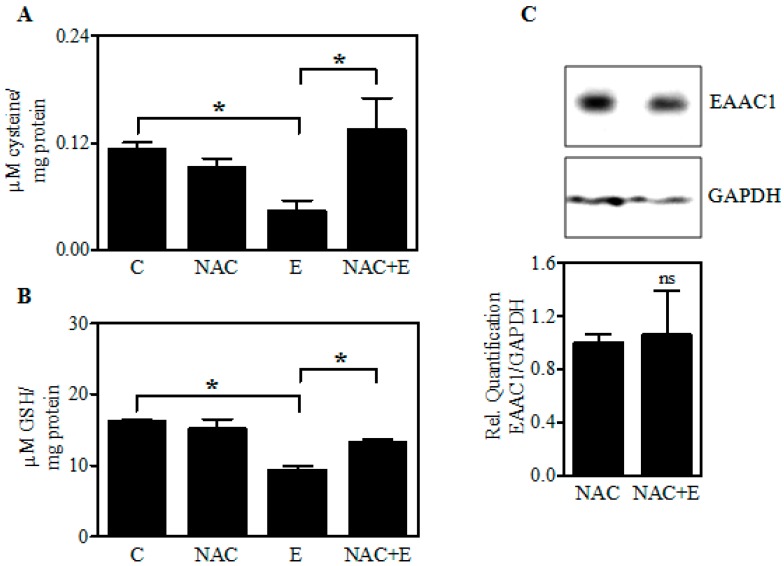
Effect of in utero *N*-acetylcysteine (NAC) pretreatment on the levels of EAAC1 protein, Cys, and GSH content. Pregnant rats (Sprague-Dawley) at embryonic day 17 were administered NAC (100 mg/kg) 1 h prior to the 1st dose of E. In total, 5 doses of E (3.5 g/kg b.wt.) or isocaloric dextrose were administered by gastric intubation at 12-h intervals. At embryonic day 19 brain cortex from embryos were dissected and processed for HPLC-based determination of Cys concentration (*n* = 3) (**A**); GSH content (*n* = 3) (**B**); Expression of EAAC1 protein in fetal brain cortices by immunoblotting (top panel) and ImageJ quantification of EAAC1 normalized to GAPDH bands (bottom panel (*n* = 3) (**C**). Values represent the mean ± SEM. * *p* < 0.05 was considered significant for ethanol alone; ns—not significant versus NAC alone treatment.

Previous studies with PCNs illustrated that pretreatment with *N*-acetylcysteine (NAC), membrane-permeable cysteine donor, mitigated the E-mediated neuronal death [9]. Figure 7 extends this to the in vivo exposure setting. Treatment of pregnant dams with NAC (100 mg/kg) for 1 h before the administration of each dose of E in the 2-day binge model significantly attenuated the E-induced Cys diminution (Figure 7A) and GSH loss (Figure 7B) in fetal brain cortices. Further, immunoblot analysis demonstrated that NAC treatment prevented the maternal E-induced EAAC1 loss in fetal brain cortices (Figure 7C). These results along with those in Figure 1 support the concept that diminution of Cys per se was responsible for E-induced GSH loss and that this could be prevented by maternal administration of NAC, a widely used cysteine prodrug, and a thiol-containing antioxidant.

## 3. Discussion

The results presented here are logical extensions of previous studies focusing on Nrf2 as a control point for the maintenance of GSH homeostasis in response to E-induced redox shifts in cultured neurons and in the developing brain. The Nrf2-related studies illustrated that the transcription factor does play a vital role in neuroprotection against E damage [7]. Yet, the neuroprotective setting remained incomplete in that GSH homeostasis was not preserved in a subpopulation of neurons in the E-exposed fetal brain [7], even in the presence of E-dependent up-regulation of Nrf2 expression. To mitigate this gap in our understanding, these new studies address a role of Cys homeostasis and their control points which could be applicable to the wide variety of neurodegenerative disorders that have been connected to OS dysregulation of the GSH redox buffer and neuron death e.g., environmental toxins, Parkinson’s disease, and Alzheimer’s disease as well as fetal alcohol spectrum disorder (FASD). 

The neuronal EAAC1 transporter is the primary supplier of Cys and thus it is a neuroprotective system separate from glutamate neurotoxicity: The importance of the EAAC1 transporter resides in the availability of Cys being requisite for neuron GSH synthesis [27,28,29] and the vast majority (90%) of neuron Cys uptake from extracellular supplies being by this excitatory amino acid transporter [26,31,32,33]. Figure 6A illustrates that a 70% knockdown of EAAC1 in PCNs produces a subsequent reduction of GSH but not GSSG (Figure 6B). The latter response causally connects a flaw in GSH synthesis to reduced EAAC1 expression and Cys transport/accrual observed on E exposure both in PCNs and fetal brain cortices.

Brain damage associated with impaired EAAC1 activity has been shown not to be connected to Glu neurotoxicity and importantly, as we have found with fetal PCNs, cell viability can be maintained by normalizing cellular GSH [5,7,15]. This was accomplished by treatment with *N*-acetylcysteine (NAC) which is a membrane-permeable Cys precursor that can easily penetrate the blood brain barrier and does not require active transport (Figure 7A,B) [44,45,46]. Notably, in congruent with our finding, NAC has been shown to provide cysteine to cells even in the absence of cysteine transport [47,48]. Interestingly, the present finding also suggests a possibility that oxidative stress can regulate EAAC1 since NAC prevented the E-induced EAAC1 loss (Figure 7C). In support of this finding, under stressful conditions, such as oxidative and chemical stress, regulation and/or alteration of EAAC1 are evident at the level of expression, activity, and its membrane trafficking [49,50,51,52]. Clinically, NAC is the widely used Cys prodrug due to its safety, tolerability, and its ability to undergo rapid hydrolysis to deliver Cys immediately following cellular entry [46,53,54].

In early-E sensitive stages of brain development, neurons maintain GSH in the absence of mature astrocytes or oligodendroglia that is generally GSH rich. Compared to other transporter subtypes such as GLAST and GLT1, EAAC1 expression precedes during early stages of brain development and is especially enriched in the cortex [55,56]. It is also expressed in mature oligodendroglia and mature astrocyte but these cells are mostly not present in the early second trimester equivalent brain [57,58,59]. Cys-related control of GSH in fetal neurons is an unexplored area. However, the possibility of such a setting in the adult brain has been suggested [60]. The present findings combined with low Cys transport activity displayed by other EAATs clearly show an E-related decrease in EAAC1 could play a key role in the diminution of Cys content (Figure 3, Figure 4 and Figure 5) [61]. Importantly, it appears that while glia can utilize both Cys and cystine for GSH synthesis, neuron utilizes only Cys [62,63]. In PCN cultures with no astrocytes, Cys uptake is mostly by EAAC1 [32,64], ASCT1 only playing a minor role [60,65]. With astrocytes present, a primary source of Cys is the γ-glutamyl cycle driven by export of GSH and its conversion to the CysGly dipeptide by astrocyte γ-glutamyl transpeptidase. We have shown that this astrocyte-mediated pathway maintains cortical neuron GSH, protecting neurons from E-mediated apoptotic death [20,66], however, this is possible only after the emergence of astrocytes which occurs at or around a late second to early third trimester period of gestation. Thus, the setting in which fetal neurons are not provided with astrocyte-mediated Cys, the former may be sensitive to E at early developmental stages concomitant with a heightened dependence on functional EAAC1-mediated Cys transport. 

We have also determined that components of the γ-glutamyl cycle are on fetal cerebral cortical neurons as well as astrocytes albeit at low levels (data not shown). In the absence of astrocytes, this could be one source of Cys for EAAC1-mediated transfer [64] as a recycling system. Placenta could be another. Given this fact, we also noted E-induced Cys downregulation is not associated with impairment in the expression of other Cysteine availability determining factors, γGT and APN showing that the access to additional extracellular cysteine may not be the limiting factor to the GSH loss. Based on our *Slc1a1* mRNA expression data, it appears that this alteration in Cys could occur due to the post-transcriptional targeting of *Slc1a1/EAAC1* by miR 96-5p and miR 26a-5p besides transcriptional regulation that has been shown to dysregulate redox homeostasis and promote neurotoxicity [49,50,67,68]. While such *Slc1a1* regulating mechanism remains to be elucidated, our results suggest a deficiency of EAAC1 transport-dependent route of neuronal Cys uptake may be one of the pivotal reasons for the E-induced GSH loss-associated toxicity. 

The present findings also point out that in utero E-induced reduction in EAAC1 could be quantitatively important during alcohol-induced redox perturbations developmentally. The genetic loss- and gain-of-function studies are underway to delineate the role of EAAC1 in alcohol developmental neurotoxicity. 

## 4. Materials and Methods

### 4.1. Materials

Fetal bovine serum (FBS) and horse serum (HS) was purchased from Atlanta Biologicals (Lawrenceville, GA, USA). Minimum Essential Media (MEM), penicillin-streptomycin and trypsin-EDTA were obtained from Gibco (Grand Island, NY, USA). Smart Pool siRNA against *Slc1a1* (EAAC1) and non-targeting siRNA pool were purchased from Dharmacon Inc., (Lafayette, CO, USA). Protein assay reagent and polyvinylidene difluoride (PVDF) membrane were from Biorad Laboratories (Hercules, CA, USA). 4–12% Bis-Tris Gel and TriZol were purchased from Invitrogen (Carlsbad, CA, USA). Antibodies for EAAC1 (Cell Signaling Technology, Beverly, MA, USA); γ-GT, APN, and GAPDH (Santa Cruz Biotechnologies, Santa Cruz, CA, USA); ACTIN were from Sigma-Aldrich (St. Louis, MO, USA). Goat anti-rabbit IgG-HRP were obtained from Cell Signaling Technology (Beverly, MA, USA). HyBlot CL autoradiography film was got from Denville Scientific (Metuchen, NJ, USA). QuantiTect reverse transcription kit was obtained from Qiagen (Valencia, CA, USA). TaqMan gene expression assays consisting of the primers and probes specific for rat *Slcla1* (Rn_00564705) and rat Gapdh (Rn_01775763) were from Applied Biosystems (Foster City, CA, USA). GSH/GSSG kit was bought from eEnzyme (Gaithersburg, MD, USA). Sulfo-NHS-SS-biotin solution and NeutrAvidin agarose beads were obtained from Thermo Fisher Scientific/Pierce (Rockford, IL, USA). Amersham ECL-Advanced Western blotting detection kit and SuperSignal West Pico chemiluminescence kit was bought from GE Healthcare Bio-Sciences (Pittsburgh, PA, USA) and Thermofisher (Rockford, IL, USA) respectively. All other reagents were purchased from Sigma-Aldrich (St. Louis, MO, USA). 

### 4.2. Primary Cortical Neuron (PCN) Cultures and Ethanol Treatment

PCNs were prepared from embryonic (E) day E16-E17 Sprague Dawley rats as described earlier [7,69]. Briefly, time-pregnant Sprague Dawley (SD) rats were euthanized by Isoflurane and cervically dislocated. Embryos were dissected; brains isolated and fetal cortices were mechanically dissociated in HBSS. Cells were then suspended in MEM containing 10% FBS and 10% HS, plated on previously coated poly-d-lysine plates and were maintained in a humidified atmosphere of 95% air and 5% CO_2_. After 24 h, the cultures were enriched for neurons by treating with 10% HS supplemented MEM media containing 5-fluoro-2′-deoxy uridine (4 mg/mL) and uridine (10 mg/mL) that inhibits the growth of astrocytes. Following 48 h, fresh media was added and PCNs were treated on 4th and 5th days of culture for transfection experiments and E exposure, respectively. This is a well-established primary neuronal culture system from our laboratory that is largely free of glia and reproducibly yielding ~95% enriched neurons [7,66,69].

Primary cultures of cortical neurons were treated with E on day 5 in vitro (DIV) at a concentration of 4 mg/mL media, a clinically relevant dose corresponding to 86 mM that is found in heavy drinkers [70,71]. Further, the E concentration used herein approximated those that were widely used to elicit a range of neurotoxic responses in various mouse and rat models [7,66,72]. E-treated plates were incubated for 24 h in an alcohol-pre-saturated incubator by placing a beaker filled with E for 24 h to prevent evaporation and maintain E concentrations in the culture media [69,73], while the control cells were maintained in the normal incubator. 

### 4.3. In Vivo Model

A very well established 2-day acute ethanol exposure animal model that mimics an alcohol binge in humans was used [7,69,74]. Briefly, Sprague Dawley rats were divided into two experimental groups and subjected to the following regimens: (1) Experimental group—This group received a total of 5 doses of 3.5 g/kg body wt, 25% *v*/*v* of ethanol (gastric intubation) starting gestational day 17 with each dose separated by 12 h interval and the last dose was given on day 19, 2 h before sacrifice to maintain blood alcohol levels (2) Control group—Pair-fed and weight matched animals receiving similar treatment regimen as the experimental group except for the intragastric intubation of iso-caloric dextrose in place of ethanol. For the NAC experiments, a single dose of NAC (100 mg /kg body wt) at embryonic day 17 was administered 1 h prior to the 1st dose of E. The NAC dose used in the current study was based on previous prenatal animal and human studies that showed NAC’s ability to normalize redox state and inflammation without any signs of toxicity [75,76,77]. All animals were maintained in accordance with Institutional Animal Care and Use Committee-approved procedures. Both isocaloric dextrose administered control and ethanol-fed dams had full access to water ad libitum, while, the pair-fed controls received the weight of standard laboratory chow consumed by the matching ethanol dam during the previous 24 h period. The gestational age of the pair-fed control and ethanol rats were staggered by a day, in order to ensure that animals from the pair-fed control received chow at the same stage of gestation as did the corresponding ethanol-treated dams. At the end of treatment, dams were decapitated and blood was collected for alcohol analysis using Analox AM1 analyzer. Fetuses were removed by cesarean section from the uterine horns and brain cortices and rest of the brain were carefully isolated and stored at −80 °C until use.

### 4.4. HPLC Based Determination of Cysteine and Total GSH

Assays of cysteine and glutathione utilized the HPLC method as described earlier with slight modifications [78]. Briefly, the sample was prepared as follows: A 50 µM of an internal standard solution of 2-mercaptopropionylgycine (MPG) in PBS was added to 250 µg of protein in a total volume of 100 µL and was vortex-mixed briefly. To this, 10 µL of 100 g/L Tris-(2-carboxyethyl)-phosphine hydrochloride (TCEP) was added, capped, vortex-mixed and incubated at room temperature for 30 min. Following this, 90 µL of TCA (100 g/L in 1 mM EDTA) was added, mixed and centrifuged at 13,000 rpm for 10 min. The TCA-precipitated supernatants were derivatized in a 0.125 M borate buffer (pH 9.5) with 4 mM EDTA containing 1.55 M NaOH and 1 g/L ammonium-7-flurobenzo-2-oxa-1,3-diazole-4-sulfonic acid (SBD-F) in borate buffer for 1 h at 60 °C. The derivatized samples were then cooled on ice for 15 min in dark until injection onto the column. The analytes, cysteine and GSH were separated on a Waters C18 guard column (3.9 mm × 20 mm; 5 µm particles, Waters Corporation, Milford, MA, USA) connected to a Waters Symmetry C18 analytical column (4.6 × 250 mm; 3.5 µm particles) at 29 °C with the mobile phase, 100 mM potassium dihydrogen phosphate (pH 2.1) and acetonitrile (92:8 *v*/*v*). The peaks of the analytes were detected at excitation and emission wavelengths of 385 nm and 515 nm, respectively with a flow rate of 1 mL/min. Retention time for cysteine and GSH was 3.6 and ~4.0–4.5 respectively. The amount of cysteine and GSH was calculated from a standard cysteine and GSH curve.

### 4.5. RNA Extraction and Real-Time qRT-PCR Analysis

Total RNA was isolated from PCNs or cerebral cortex using the TRIzol reagent according to the manufacturer’s recommendations (Invitrogen, Carlsbad, CA, USA). Following genomic DNA elimination, 1.5 µg of total RNA was reverse-transcribed using the QuantiTect reverse transcription kit. 1/10th of the cDNA in a final volume of 20 µL containing 10 µL of TaqMan Universal Master Mix (Applied Biosystems, Bedford, MA, USA), 20 pmol of the respective primer/probe mix was used in a real-time RT-PCR reaction to determine the mRNA expression for *Slc1a1* (*Eaac1*), and *Gapdh* using an initial denaturation step of 95 °C for 30 s followed by 40 PCR cycles at 95 °C for 5 s and 60 °C for 30 s. The expression of *Slc1a1* was determined relative to *Gapdh* as an internal control and the relative fold change in the mRNA expression was calculated using the 2^−ΔΔ*C*t^, where Δ*C*t = *C*t*_Slc1a1_* − *C*t*_Gapdh_* and ΔΔ*C*t = Δ*C*t_treated condition_ − Δ*C*t_untreated condition_.

### 4.6. Reverse Transcription-PCR Analysis

1/10th of cDNA prepared as earlier was used in a PCR reaction using primers specific for rat *γGt*-NM_053840.2 (For: 5′-CTCTGCATCTGGCTACCCAC-3′; Rev: 5′-GGATGCTGGGTTGGAA-3′); rat *ApN* GenBank: M26710.1 (For: 5′-GCCCATTCCGTATCTCAAAA-3′; Rev: 5′-AAGTAGGCGAAGAGGGGTGT-3′) [79] and rat *Gapdh* (For: 5′-AGACAGCCGCATCTTCTTGT-3′; Rev: 5′-TACTCAGCACCAGCATCACC-3′). After an initial denaturation at 95 °C for 3 min, PCR specific for *γGt* and *ApN* was performed in a 25 µL-reaction volume for 35 cycles under the following conditions: 95 °C for 30 s, 53 °C for 30 s, 72 °C for 60 s, and finally an extension at 72 °C for 5 min. *Gapdh* cycling parameters were the same except for the annealing temperature which is 55 °C for 30 s. Aliquots of the PCR product was run on 1% agarose gel and the products for *γGt*, *ApN*, and *Gapdh* were visualized at 419 bp, 438 bp, and 323 bp respectively by ethidium bromide staining using UVP gel documentation system. The images were photographed and quantified using NIH Image J software (v1.42q, Bethesda, MD, USA). 

### 4.7. Gel Electrophoresis and Immunoblotting

PCNs or cerebral cortices were lysed in radio-immunoprecipitation assay (RIPA) buffer containing protease inhibitor cocktail (Sigma-Aldrich, St. Louis, MO, USA) at 4 °C, sonicated (Sonics, vibra-cell ultrasonic processor) for 5 s, centrifuged at 15,000× *g* for 15 min at 4 °C. The clarified supernatants were estimated for protein concentration and equal amounts of cellular protein were loaded on a 4–12% Bis-Tris gel. Sodium dodecyl sulfate polyacrylamide gel electrophoresis (SDS-PAGE)-separated proteins were electro-transferred onto a PVDF membrane and blocked with 5% nonfat dry milk powder in PBST for 1 h. The membranes were then incubated with primary antibodies against EAAC1, γ-GT, APN or GAPDH or ACTIN for 3 h or overnight at 4 °C as previously described [69,80] and subsequently washed with PBST for 3 times. Later the membranes were incubated with anti-rabbit IgG secondary antibody conjugated with horseradish peroxidase (1:5000 or 1:10,000) for 1 h at room temperature, washed with PBST for 5 min × 5 times each, and subjected to ECL-chemiluminescence to detect the bands of proteins. EAAC1 bands detected using Amersham ECL-Advanced Western blotting detection kit, and the GAPDH or ACTIN signals detected using SuperSignal West Pico chemiluminescence kit were captured onto an autoradiography film and scanned using Adobe Photoshop CS2 (v9.0, Mountain View, CA, USA). The intensity of EAAC1 bands was quantified using NIH Image J and normalized to GAPDH or ACTIN band intensity.

### 4.8. In Vitro Biotinylation Assay for Cell Surface Determination of EAAC1

In vitro biotinylation assay to determine the cell surface changes of EAAC1 was performed as previously described with slight modification [81]. In brief, PCNs at DIV5 with or without E were washed in cold PBS/CaCl_2_/MgCl_2_, pH 8.0 and were incubated in fresh cold PBS containing 0.5 mg/mL sulfo-NHS-SS-biotin for 30 min. After extensive washes in cold PBS, the nonreacted sulfo-NHS-SS-biotin was quenched by incubating the cells in 50 mM glycine in PBS for 15 min with every 5 min replacing with fresh quenching solution. Cells were then lysed in IP buffer (PBS, 5 mM EDTA and EGTA, 10 mM sodium pyrophosphate, 1 mM beta-glycerophosphate, 1 mM NaVO_3_, 1% Triton and protease inhibitors). Protein was measured in the clarified supernatants and 500 µg of protein from each sample was incubated with pre-equilibrated NeutrAvidin agarose at 4 °C with overnight rotation. After incubation, samples were centrifuged at 4 °C, 5000 rpm for 1 min, supernatant removed and the NeutrAvidin beads bound with cell surface proteins (biotinylated) were washed three times in IP buffer. The beads were boiled in sample buffer for SDS-PAGE and Western blot analysis was performed for EAAC1. ACTIN expression was performed in the supernatant containing the intracellular proteins (unbiotinylated) and the biotinylated EAAC1 values were normalized to intracellular ACTIN. 

### 4.9. Small Interfering RNA (siRNA) Transfection

100 nM of siGenome smartpool mix of four *Slc1a1* (*EAAC1*) specific siRNAs or non-targeting siRNA pool were transfected into 4DIV neurons using siPort Amine (Ambion). EAAC1 silencing was observed by Western analysis after 48 h.

### 4.10. Fluorimetry Based Assay for Reduced and Oxidized Glutathione (GSH/GSSG)

GSH/GSSG determinations in siRNA-transfected PCNs or fetal brain cortices were performed according to the manufacturer’s instructions (eEnzyme, Gaithersburg, MD, USA). Briefly, the lysates were prepared from either fetal brain cortices or *Slc1a1/EAAC1*-silenced neurons post 48 h of transfection using cell lysis buffer, centrifuged at 2500 rpm for 15 min and the supernatant collected. For measuring GSH and Total GSH (TGSH), a separate aliquot of the test lysate is mixed with GSH Assay Mixture (GAM) containing a non-fluorescent, Thiolite GreenWS dye and Total GSH Assay Mixture (TGAM) containing a mixture of GSSG probe and GAM solution into a black 96-well microplate. The samples were incubated at room temperature for 30 min by protecting from light followed by which the fluorescence was monitored at Ex/Em = 490/520 nm using a fluorescence plate reader. Assay buffer only served as blank. Using serially diluted GSH and GSSG standards that were run in parallel, the test values were calculated and plotted. The GSSG concentration was estimated using the following formula: [GSSG] = ([Total GSH] − [GSH])/2.

### 4.11. Statistical Analysis

Data are presented as mean ± S.E.M. Statistical differences were determined using one-way ANOVA followed by Student-Newman-Keuls post-hoc analysis when experiments involved more than two groups. Student’s *t*-test was used for experiments involving only two groups. The analysis was carried out using GraphPad software. *p* < 0.05 was considered as statistically significant.

## Abbreviations

GSH	Reduced Glutathione
GSSG	Oxidized Glutathione
Cys	Cysteine
EAAC1/EAAT3/Slc1a1	Excitatory Amino Acid Carrier 1
γGT	Gamma-glutamyl Transpeptidase
APN	Amionopeptidase N

## References

[1] Bearer C.F., Swick A.R., O’Riordan M.A., Cheng G. (1999). Ethanol inhibits L1-mediated neurite outgrowth in postnatal rat cerebellar granule cells. J. Biol. Chem..

[2] Goodlett C.R., Thomas J.D., West J.R. (1991). Long-term deficits in cerebellar growth and rotarod performance of rats following “binge-like” alcohol exposure during the neonatal brain growth spurt. Neurotoxicol. Teratol..

[3] Kodali V.N., Jacobson J.L., Lindinger N.M., Dodge N.C., Molteno C.D., Meintjes E.M., Jacobson S.W. (2017). Differential recruitment of brain regions during response inhibition in children prenatally exposed to alcohol. Alcohol. Clin. Exp. Res..

[4] Mooney S.M., Napper R.M. (2005). Early postnatal exposure to alcohol reduces the number of neurons in the occipital but not the parietal cortex of the rat. Alcohol. Clin. Exp. Res..

[5] Maffi S.K., Rathinam M.L., Cherian P.P., Pate W., Hamby-Mason R., Schenker S., Henderson G.I. (2008). Glutathione content as a potential mediator of the vulnerability of cultured fetal cortical neurons to ethanol-induced apoptosis. J. Neurosci. Res..

[6] Maier S.E., West J.R. (2001). Regional differences in cell loss associated with binge-like alcohol exposure during the first two trimesters equivalent in the rat. Alcohol.

[7] Narasimhan M., Mahimainathan L., Rathinam M.L., Riar A.K., Henderson G.I. (2011). Overexpression of Nrf2 protects cerebral cortical neurons from ethanol-induced apoptotic death. Mol. Pharmacol..

[8] Ramachandran V., Perez A., Chen J., Senthil D., Schenker S., Henderson G.I. (2001). In utero ethanol exposure causes mitochondrial dysfunction, which can result in apoptotic cell death in fetal brain: A potential role for 4-hydroxynonenal. Alcohol. Clin. Exp. Res..

[9] Ramachandran V., Watts L.T., Maffi S.K., Chen J., Schenker S., Henderson G. (2003). Ethanol-induced oxidative stress precedes mitochondrially mediated apoptotic death of cultured fetal cortical neurons. J. Neurosci. Res..

[10] Young C., Olney J.W. (2006). Neuroapoptosis in the infant mouse brain triggered by a transient small increase in blood alcohol concentration. Neurobiol. Dis..

[11] Young C., Roth K.A., Klocke B.J., West T., Holtzman D.M., Labruyere J., Qin Y.Q., Dikranian K., Olney J.W. (2005). Role of caspase-3 in ethanol-induced developmental neurodegeneration. Neurobiol. Dis..

[12] Heaton M.B., Paiva M., Madorsky I., Shaw G. (2003). Ethanol effects on neonatal rat cortex: Comparative analyses of neurotrophic factors, apoptosis-related proteins, and oxidative processes during vulnerable and resistant periods. Brain Res. Dev. Brain Res..

[13] Heaton M.B., Paiva M., Mayer J., Miller R. (2002). Ethanol-mediated generation of reactive oxygen species in developing rat cerebellum. Neurosci. Lett..

[14] Narasimhan M., Rathinam M., Patel D., Henderson G., Mahimainathan L. (2012). Astrocytes prevent ethanol induced apoptosis of Nrf2 depleted neurons by maintaining GSH homeostasis. Open J. Apoptosis.

[15] Hansen J.M., Harris C. (2013). Redox control of teratogenesis. Reprod. Toxicol..

[16] Harris C., Shuster D.Z., Roman Gomez R., Sant K.E., Reed M.S., Pohl J., Hansen J.M. (2013). Inhibition of glutathione biosynthesis alters compartmental redox status and the thiol proteome in organogenesis-stage rat conceptuses. Free Radic. Biol. Med..

[17] Jones D.P. (2008). Radical-free biology of oxidative stress. Am. J. Physiol. Cell Physiol..

[18] Schafer F.Q., Buettner G.R. (2001). Redox environment of the cell as viewed through the redox state of the glutathione disulfide/glutathione couple. Free Radic. Biol. Med..

[19] Hamby-Mason R.L., Mason P.A., Schenker S., Henderson G.I. (1998). Histochemical method for localization of hydrogen peroxide and oxygen radicals in the intact neonatal brain. Methods Find. Exp. Clin. Pharmacol..

[20] Watts L.T., Rathinam M.L., Schenker S., Henderson G.I. (2005). Astrocytes protect neurons from ethanol-induced oxidative stress and apoptotic death. J. Neurosci. Res..

[21] Dennery P.A. (2007). Effects of oxidative stress on embryonic development. Birth Defects Res. C Embryo Today.

[22] Fantel A.G. (1996). Reactive oxygen species in developmental toxicity: Review and hypothesis. Teratology.

[23] Wells P.G., Kim P.M., Laposa R.R., Nicol C.J., Parman T., Winn L.M. (1997). Oxidative damage in chemical teratogenesis. Mutat. Res..

[24] Wells P.G., McCallum G.P., Chen C.S., Henderson J.T., Lee C.J., Perstin J., Preston T.J., Wiley M.J., Wong A.W. (2009). Oxidative stress in developmental origins of disease: Teratogenesis, neurodevelopmental deficits, and cancer. Toxicol. Sci..

[25] Ma Q. (2013). Role of nrf2 in oxidative stress and toxicity. Annu. Rev. Pharmacol. Toxicol..

[26] Aoyama K., Watabe M., Nakaki T. (2008). Regulation of neuronal glutathione synthesis. J. Pharmacol. Sci..

[27] Lu S.C. (2009). Regulation of glutathione synthesis. Mol. Asp. Med..

[28] Huang C.S., Moore W.R., Meister A. (1988). On the active site thiol of γ-glutamylcysteine synthetase: Relationships to catalysis, inhibition, and regulation. Proc. Natl. Acad. Sci. USA.

[29] Meister A., Anderson M.E. (1983). Glutathione. Annu. Rev. Biochem..

[30] Go Y.M., Jones D.P. (2013). Thiol/disulfide redox states in signaling and sensing. Crit. Rev. Biochem. Mol. Biol..

[31] Aoyama K., Nakaki T. (2013). Neuroprotective properties of the excitatory amino acid carrier 1 (EAAC1). Amino Acids.

[32] Aoyama K., Watabe M., Nakaki T. (2012). Modulation of neuronal glutathione synthesis by EAAC1 and its interacting protein GTRAP3-18. Amino Acids.

[33] Johnson W.M., Wilson-Delfosse A.L., Mieyal J.J. (2012). Dysregulation of glutathione homeostasis in neurodegenerative diseases. Nutrients.

[34] Aoyama K., Suh S.W., Hamby A.M., Liu J., Chan W.Y., Chen Y., Swanson R.A. (2006). Neuronal glutathione deficiency and age-dependent neurodegeneration in the EAAC1 deficient mouse. Nat. Neurosci..

[35] Zerangue N., Kavanaugh M.P. (1996). Interaction of l-cysteine with a human excitatory amino acid transporter. J. Physiol..

[36] Berman A.E., Chan W.Y., Brennan A.M., Reyes R.C., Adler B.L., Suh S.W., Kauppinen T.M., Edling Y., Swanson R.A. (2011). *N*-acetylcysteine prevents loss of dopaminergic neurons in the EAAC1-/- mouse. Ann. Neurol..

[37] Cao L., Li L., Zuo Z. (2012). N-acetylcysteine reverses existing cognitive impairment and increased oxidative stress in glutamate transporter type 3 deficient mice. Neuroscience.

[38] Gressens P., Lammens M., Picard J.J., Evrard P. (1992). Ethanol-induced disturbances of gliogenesis and neuronogenesis in the developing murine brain: An in vitro and in vivo immunohistochemical and ultrastructural study. Alcohol Alcohol..

[39] Won S.J., Yoo B.H., Brennan A.M., Shin B.S., Kauppinen T.M., Berman A.E., Swanson R.A., Suh S.W. (2010). EAAC1 gene deletion alters zinc homeostasis and exacerbates neuronal injury after transient cerebral ischemia. J. Neurosci..

[40] Escartin C., Won S.J., Malgorn C., Auregan G., Berman A.E., Chen P.C., Deglon N., Johnson J.A., Suh S.W., Swanson R.A. (2011). Nuclear factor erythroid 2-related factor 2 facilitates neuronal glutathione synthesis by upregulating neuronal excitatory amino acid transporter 3 expression. J. Neurosci..

[41] Fournier K.M., Gonzalez M.I., Robinson M.B. (2004). Rapid trafficking of the neuronal glutamate transporter, EAAC1: Evidence for distinct trafficking pathways differentially regulated by protein kinase C and platelet-derived growth factor. J. Biol. Chem..

[42] Gonzalez M.I., Susarla B.T., Fournier K.M., Sheldon A.L., Robinson M.B. (2007). Constitutive endocytosis and recycling of the neuronal glutamate transporter, excitatory amino acid carrier 1. J. Neurochem..

[43] Li X., Valencia A., Sapp E., Masso N., Alexander J., Reeves P., Kegel K.B., Aronin N., Difiglia M. (2010). Aberrant Rab11-dependent trafficking of the neuronal glutamate transporter EAAC1 causes oxidative stress and cell death in Huntington’s disease. J. Neurosci..

[44] Farr S.A., Poon H.F., Dogrukol-Ak D., Drake J., Banks W.A., Eyerman E., Butterfield D.A., Morley J.E. (2003). The antioxidants alpha-lipoic acid and *N*-acetylcysteine reverse memory impairment and brain oxidative stress in aged SAMP8 mice. J. Neurochem..

[45] Katz M., Won S.J., Park Y., Orr A., Jones D.P., Swanson R.A., Glass G.A. (2015). Cerebrospinal fluid concentrations of *N*-acetylcysteine after oral administration in Parkinson’s disease. Parkinsonism Relat. Disord..

[46] Sen C.K. (1997). Nutritional biochemistry of cellular glutathione. J. Nutr. Biochem..

[47] Mazor D., Golan E., Philip V., Katz M., Jafe A., Ben-Zvi Z., Meyerstein N. (1996). Red blood cell permeability to thiol compounds following oxidative stress. Eur. J. Haematol..

[48] Parsons J.L., Chipman J.K. (2000). The role of glutathione in DNA damage by potassium bromate in vitro. Mutagenesis.

[49] Guo M., Cao D., Zhu S., Fu G., Wu Q., Liang J., Cao M. (2015). Chronic exposure to morphine decreases the expression of EAAT3 via opioid receptors in hippocampal neurons. Brain Res..

[50] Harrington E.P., Moddel G., Najm I.M., Baraban S.C. (2007). Altered glutamate receptor—Transporter expression and spontaneous seizures in rats exposed to methylazoxymethanol in utero. Epilepsia.

[51] Liu Y., Vidensky S., Ruggiero A.M., Maier S., Sitte H.H., Rothstein J.D. (2008). Reticulon RTN2B regulates trafficking and function of neuronal glutamate transporter EAAC1. J. Biol. Chem..

[52] Yun J.Y., Park K.S., Kim J.H., Do S.H., Zuo Z. (2007). Propofol reverses oxidative stress-attenuated glutamate transporter EAAT3 activity: Evidence of protein kinase C involvement. Eur. J. Pharmacol..

[53] Borgstrom L., Kagedal B., Paulsen O. (1986). Pharmacokinetics of N-acetylcysteine in man. Eur. J. Clin. Pharmacol..

[54] Olsson B., Johansson M., Gabrielsson J., Bolme P. (1988). Pharmacokinetics and bioavailability of reduced and oxidized *N*-acetylcysteine. Eur. J. Clin. Pharmacol..

[55] Furuta A., Rothstein J.D., Martin L.J. (1997). Glutamate transporter protein subtypes are expressed differentially during rat CNS development. J. Neurosci..

[56] Sims K.D., Robinson M.B. (1999). Expression Patterns and Regulation of Glutamate Transporters in the Developing and Adult Nervous System. Crit. Rev. Neurobiol..

[57] Freeman M.R. (2010). Specification and morphogenesis of astrocytes. Science.

[58] McKenzie I.A., Ohayon D., Li H., de Faria J.P., Emery B., Tohyama K., Richardson W.D. (2014). Motor skill learning requires active central myelination. Science.

[59] Qian X., Shen Q., Goderie S.K., He W., Capela A., Davis A.A., Temple S. (2000). Timing of CNS cell generation: A programmed sequence of neuron and glial cell production from isolated murine cortical stem cells. Neuron.

[60] Shanker G., Allen J.W., Mutkus L.A., Aschner M. (2001). The uptake of cysteine in cultured primary astrocytes and neurons. Brain Res..

[61] Watts S.D., Torres-Salazar D., Divito C.B., Amara S.G. (2014). Cysteine transport through excitatory amino acid transporter 3 (EAAT3). PLoS ONE.

[62] Kranich O., Hamprecht B., Dringen R. (1996). Different preferences in the utilization of amino acids for glutathione synthesis in cultured neurons and astroglial cells derived from rat brain. Neurosci. Lett..

[63] Wang X.F., Cynader M.S. (2000). Astrocytes provide cysteine to neurons by releasing glutathione. J. Neurochem..

[64] Nieoullon A., Canolle B., Masmejean F., Guillet B., Pisano P., Lortet S. (2006). The neuronal excitatory amino acid transporter EAAC1/EAAT3: Does it represent a major actor at the brain excitatory synapse?. J. Neurochem..

[65] Chen Y., Swanson R.A. (2003). The glutamate transporters EAAT2 and EAAT3 mediate cysteine uptake in cortical neuron cultures. J. Neurochem..

[66] Rathinam M.L., Watts L.T., Stark A.A., Mahimainathan L., Stewart J., Schenker S., Henderson G.I. (2006). Astrocyte control of fetal cortical neuron glutathione homeostasis: Up-regulation by ethanol. J. Neurochem..

[67] Kinoshita C., Aoyama K., Matsumura N., Kikuchi-Utsumi K., Watabe M., Nakaki T. (2014). Rhythmic oscillations of the microRNA miR-96-5p play a neuroprotective role by indirectly regulating glutathione levels. Nat. Commun..

[68] Potenza N., Mosca N., Mondola P., Damiano S., Russo A., de Felice B. (2017). Human miR-26a-5p regulates the glutamate transporter SLC1A1 (EAAT3) expression. Relevance in multiple sclerosis. Biochim. Biophys. Acta.

[69] Narasimhan M., Rathinam M., Riar A., Patel D., Mummidi S., Yang H.S., Colburn N.H., Henderson G.I., Mahimainathan L. (2013). Programmed cell death 4 (PDCD4): A novel player in ethanol-mediated suppression of protein translation in primary cortical neurons and developing cerebral cortex. Alcohol. Clin. Exp. Res..

[70] Jones A.W. (1999). The drunkest drinking driver in Sweden: Blood alcohol concentration 0.545% *w*/*v*. J. Stud. Alcohol.

[71] Page A., Paoli P.P., Hill S.J., Howarth R., Wu R., Kweon S.M., French J., White S., Tsukamoto H., Mann D.A. (2015). Alcohol directly stimulates epigenetic modifications in hepatic stellate cells. J. Hepatol..

[72] Dong J., Sulik K.K., Chen S.Y. (2008). Nrf2-mediated transcriptional induction of antioxidant response in mouse embryos exposed to ethanol in vivo: Implications for the prevention of fetal alcohol spectrum disorders. Antioxid. Redox Signal..

[73] Riar A.K., Narasimhan M., Rathinam M.L., Henderson G.I., Mahimainathan L. (2016). Ethanol induces cytostasis of cortical basal progenitors. J. Biomed. Sci..

[74] Henderson G.I., Devi B.G., Perez A., Schenker S. (1995). In utero ethanol exposure elicits oxidative stress in the rat fetus. Alcohol. Clin. Exp. Res..

[75] Xu D.X., Chen Y.H., Wang H., Zhao L., Wang J.P., Wei W. (2005). Effect of *N*-acetylcysteine on lipopolysaccharide-induced intra-uterine fetal death and intra-uterine growth retardation in mice. Toxicol. Sci..

[76] Beloosesky R., Ginsberg Y., Khatib N., Maravi N., Ross M.G., Itskovitz-Eldor J., Weiner Z. (2013). Prophylactic maternal N-acetylcysteine in rats prevents maternal inflammation-induced offspring cerebral injury shown on magnetic resonance imaging. Am. J. Obstet. Gyneol..

[77] Jenkins D.D., Wiest D.B., Mulvihill D.M., Hlavacek A.M., Majstoravich S.J., Brown T.R., Taylor J.J., Buckley J.R., Turner R.P., Rollins L.G. (2016). Fetal and neonatal effects of *N*-acetylcysteine when used for neuroprotection in maternal chorioamnionitis. J. Pediatr..

[78] Nolin T.D., McMenamin M.E., Himmelfarb J. (2007). Simultaneous determination of total homocysteine, cysteine, cysteinylglycine, and glutathione in human plasma by high-performance liquid chromatography: Application to studies of oxidative stress. J. Chromatogr. B Anal. Technol. Biomed. Life Sci..

[79] Dringen R., Gutterer J.M., Gros C., Hirrlinger J. (2001). Aminopeptidase N mediates the utilization of the GSH precursor CysGly by cultured neurons. J. Neurosci. Res..

[80] Riar A.K., Narasimhan M., Rathinam M.L., Vedpathak D., Mummidi S., Henderson G.I., Mahimainathan L. (2014). Ethanol-induced transcriptional activation of programmed cell death 4 (Pdcd4) is mediated by GSK-3beta signaling in rat cortical neuroblasts. PLoS ONE.

[81] Huang G.N. (2012). Biotinylation of Cell Surface Proteins. Bio-protocol.

